# Plasma Genotyping at the Time of Diagnostic Tissue Biopsy Decreases Time-to-Treatment in Patients With Advanced NSCLC—Results From a Prospective Pilot Study

**DOI:** 10.1016/j.jtocrr.2022.100301

**Published:** 2022-03-08

**Authors:** Jeffrey C. Thompson, Charu Aggarwal, Janeline Wong, Vivek Nimgaonkar, Wei-Ting Hwang, Michelle Andronov, David M. Dibardino, Christoph T. Hutchinson, Kevin C. Ma, Anthony Lanfranco, Edmund Moon, Andrew R. Haas, Aditi P. Singh, Christine A. Ciunci, Melina Marmarelis, Christopher D’Avella, Justine V. Cohen, Joshua M. Bauml, Roger B. Cohen, Corey J. Langer, Anil Vachani, Erica L. Carpenter

**Affiliations:** a Thoracic Oncology Group, Division of Pulmonary, Allergy, and Critical Care Medicine, Department of Medicine, Perelman School of Medicine, University of Pennsylvania, Philadelphia, Pennsylvania; b Division of Hematology-Oncology, Department of Medicine, Perelman School of Medicine, University of Pennsylvania, Philadelphia, Pennsylvania; c Perelman School of Medicine, University of Pennsylvania, Philadelphia, Pennsylvania; d Department of Biostatistics, Epidemiology and Informatics, Perelman School of Medicine, University of Pennsylvania, Philadelphia, Pennsylvania

**Keywords:** Lung cancer, Precision medicine, Lung cancer genomics, Circulating tumor DNA, Multidisciplinary

## Abstract

**Introduction:**

The availability of targeted therapies has transformed the management of advanced NSCLC; however, most patients do not undergo guideline-recommended tumor genotyping. The impact of plasma-based next-generation sequencing (NGS) performed simultaneously with diagnostic biopsy in suspected advanced NSCLC has largely been unexplored.

**Methods:**

We performed a prospective cohort study of patients with suspected advanced lung cancer on the basis of cross-sectional imaging results. Blood from the time of biopsy was sequenced using a commercially available 74-gene panel. The primary outcome measure was time to first-line systemic treatment compared with a retrospective cohort of consecutive patients with advanced NSCLC with reflex tissue NGS.

**Results:**

We analyzed the NGS results from 110 patients with newly diagnosed advanced NSCLC: cohorts 1 and 2 included 55 patients each and were well balanced regarding baseline demographics. In cohort 1, plasma NGS identified therapeutically informative driver mutations in 32 patients (58%) (13 *KRAS* [five *KRAS G12C*], 13 *EGFR*, two *ERRB2*, two *MET*, one *BRAF*, one *RET*). The NGS results were available before the first oncology visit in 85% of cohort 1 versus 9% in cohort 2 (*p* < 0.0001), with more cohort 1 patients receiving a guideline-concordant treatment recommendation at this visit (74% versus 46%, *p* = 0.005). Time-to-treatment was significantly shorter in cohort 1 compared with cohort 2 (12 versus 20 d, *p* = 0.003), with a shorter time-to-treatment in patients with specific driver mutations (10 versus 19 d, *p* = 0.001).

**Conclusions:**

Plasma-based NGS performed at the time of diagnostic biopsy in patients with suspected advanced NSCLC is associated with decreased time-to-treatment compared with usual care.

## Introduction

With the continued expansion of approved targeted therapies for patients with advanced NSCLC, molecular testing for actionable mutations is crucial to delivering personalized therapy; however, most patients do not have guideline-concordant genotyping performed.^1^, ^2^, ^3^ Barriers include insufficient tissue specimens, inaccessibility of molecular testing, and long turnaround times for results.^4^ Plasma-based genotyping using circulating cell-free tumor DNA is highly concordant with tissue sequencing with rapid turnaround times and is increasingly used in clinical practice to overcome some of these challenges.^1^^,^^5^

Molecular profiling, whether tissue- or plasma-based, is often initiated at the first medical oncology visit. Before this visit, patients have typically undergone an extended workup beginning with identification of a radiographic abnormality and subsequent subspecialty referral for biopsy and confirmation of lung cancer followed by referral to medical oncology and other cancer specialists. This process may take several weeks. The timeliness of biomarker testing is essential to avoid treatment delays or inappropriate treatment assignments if therapeutic decisions are made before availability of results.^6^ We hypothesized that performing plasma-based genotyping at the time of diagnostic biopsy in cases of suspected advanced NSCLC would reduce time-to-treatment and improve the likelihood that treatment decisions were made with knowledge regarding all guideline-recommended targets evaluated.

## Materials and Methods

This single-center, prospective cohort study was conducted at the University of Pennsylvania between September 2019 and April 2021. Eligible patients had suspected advanced (stage IIIB-IV) NSCLC by imaging and underwent evaluation by an interventional pulmonologist for diagnostic biopsy. Patients with other active malignancies were excluded. Blood was collected before the time of diagnostic biopsy and sequenced using the Guardant360 74-gene next-generation sequencing (NGS) assay.^7^ Biopsy specimens with nonsquamous NSCLC were reflexed for sequencing using a 152-gene NGS panel on the basis of institutional standard of care (SOC). The primary end point was time-to-treatment, defined as the time from diagnostic tissue biopsy to selection of a first-line systemic therapy.

A contemporary historical cohort of consecutive patients with advanced NSCLC that underwent conventional reflex tissue NGS was identified in the 12 months before prospective enrollment (September 2018–August 2019) (cohort 2). Patients in the historical cohort were each evaluated by an interventional pulmonologist, underwent biopsy confirmation of advanced NSCLC with reflex tissue NGS, and received oncologic care at our center. The study was approved by the institutional review board, and all prospectively enrolled patients provided written informed consent. All statistical analyses were two-sided and performed using Stata version 15.1 (College Station, TX).

## Results

**Figure 1:**
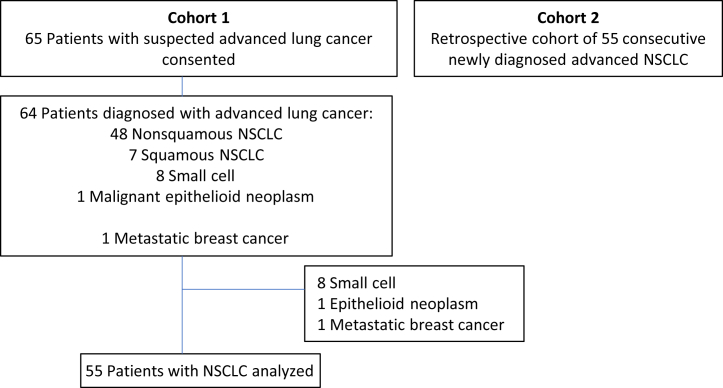
Cohort flowchart.

A total of 65 patients with suspected advanced NSCLC were prospectively enrolled and completed plasma NGS at the time of biopsy. After excluding patients without NSCLC (n = 10), 55 patients with advanced NSCLC were included in the final analysis (cohort 1). Cohort 2 included 55 consecutive patients with advanced NSCLC who received SOC reflex tissue genotyping (Fig. 1). The cohorts were well balanced with regard to variables illustrated in Supplementary Table 1. Plasma NGS was completed for all patients in cohort 1. A total of 16 patients (29%) in cohort 2 underwent plasma NGS ordered by their medical oncologist as part of SOC subsequent to the time of diagnostic biopsy. The number of patients identified to have a driver mutation by tissue or liquid biopsy was similar in both cohorts (62% versus 64%, *p* = 0.84, Pearson’s chi-square; Supplementary Table 2).

**Figure 2:**
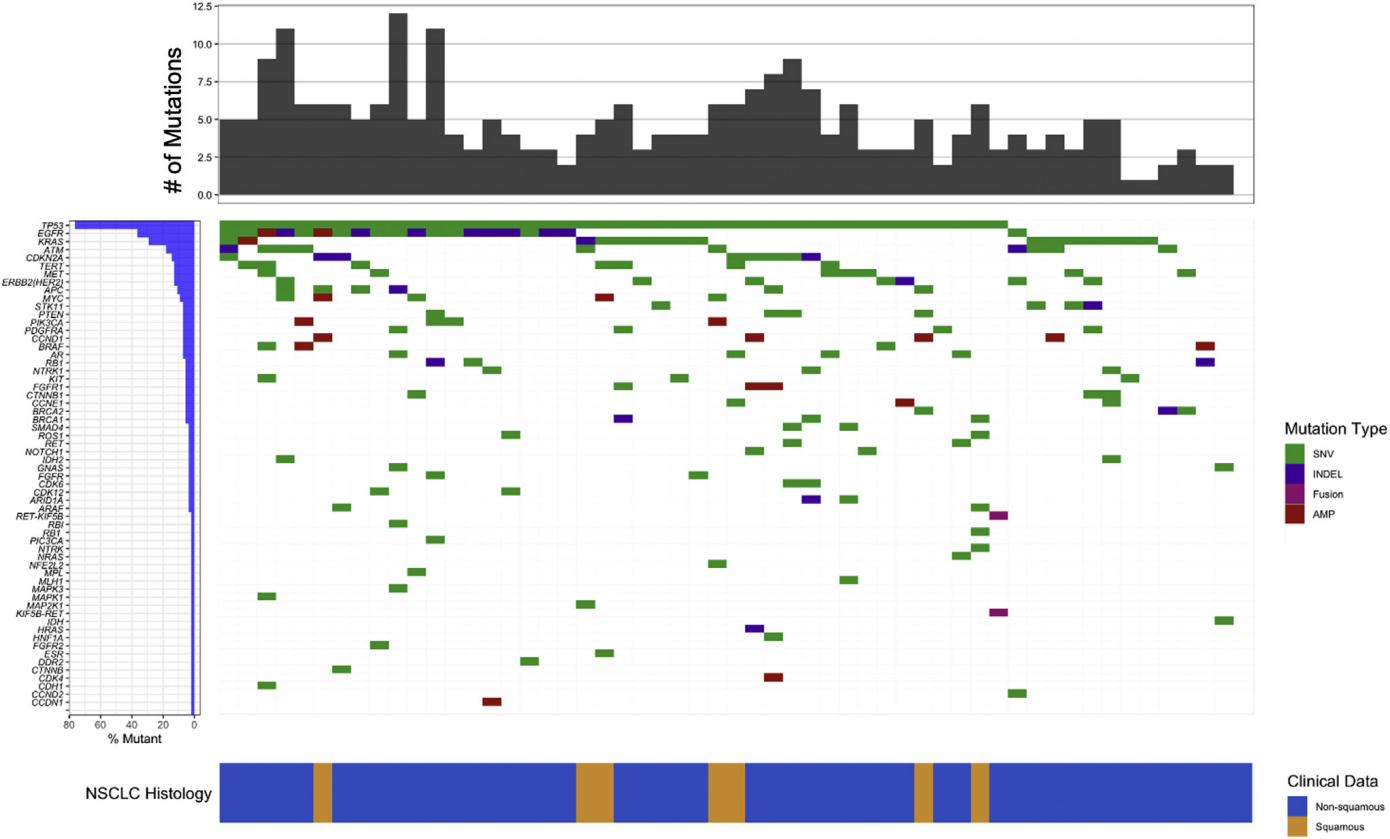
Plasma genotyping mutational profile. Plasma genotyping results revealing SNVs (green), INDELs (purple), fusions (magenta), and gene amplifications (red) detected in each gene for the 55 patients in cohort 1. Each row indicates a gene for which one or more patients had a mutation detected, with rows ordered from top to bottom on the basis of decreasing prevalence of mutations. Number of variants detected in each patient is represented by the height of the top gray bars. The row at the bottom indicates NSCLC histology with nonsquamous (blue) and squamous (gold) histologies. #, number; AMP, amplification; INDEL, insertion/deletion; SNV, single-nucleotide variant.

**Figure 3:**
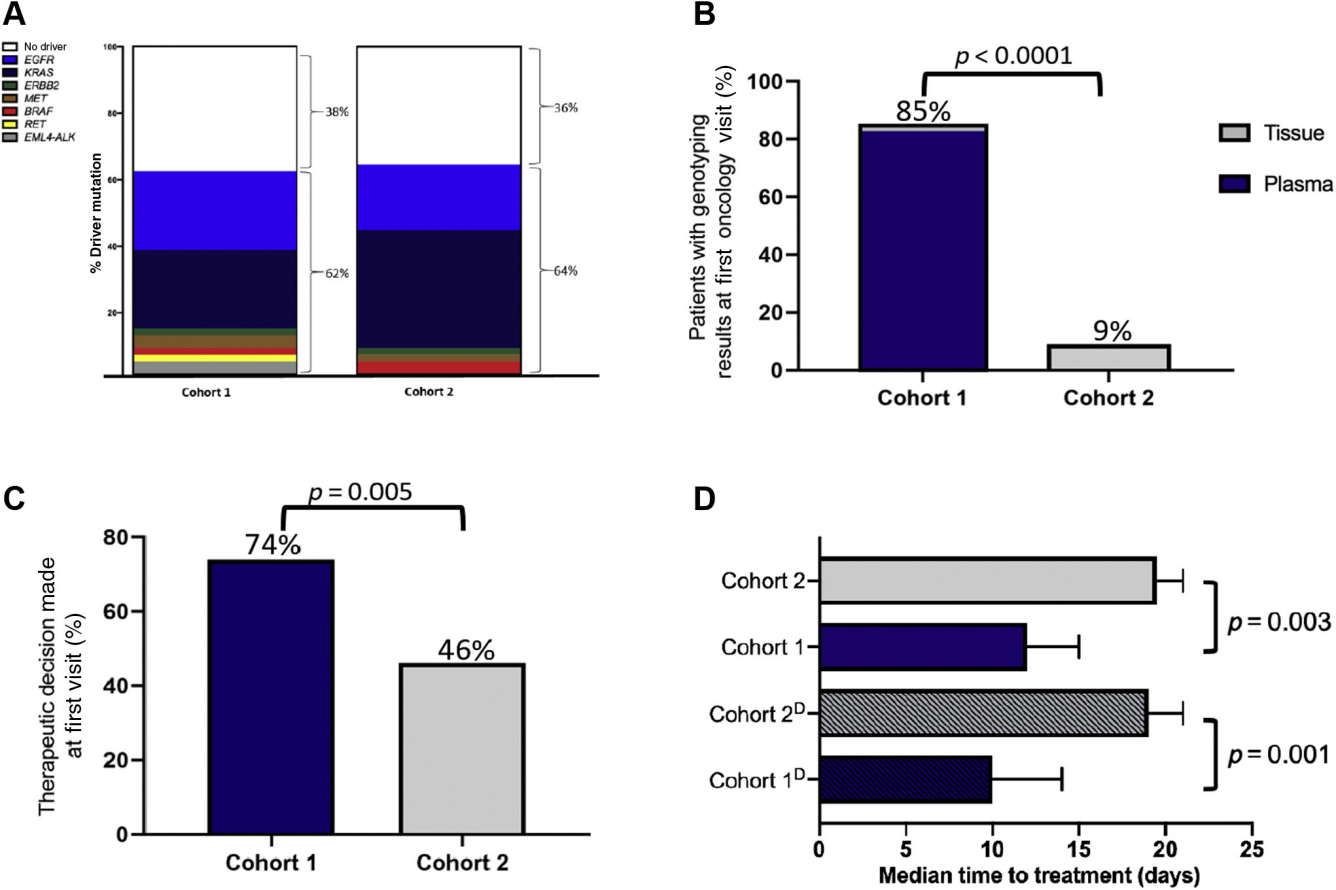
Impact of plasma NGS at time of biopsy on time-to-treatment. (*A*) Comparison of the total number of driver mutations detected in both cohorts. (*B*) Comparison of NGS results available at the first oncology visit between cohorts. Tissue results available depicted in gray and plasma NGS results depicted in blue. (*C*) Percentage of patients receiving a specific treatment recommendation at the first oncology visit. (*D*) Median time-to-time treatment between cohorts. Cohort^D^ depicts patients with a therapeutically informative driver mutation detected. NGS, next-generation sequencing.

In cohort 1, at least one somatic variant was detected in 54 prebiopsy plasma NGS specimens (98%) (Fig. 2 and Supplementary Table 3). Plasma NGS identified a therapeutically informative driver mutation in 32 patients (58%) (13 *KRAS* [five G12*C*], 13 *EGFR*, two *ERRB2* [*HER2*], two *MET*, one *BRAF*, and one *RET*) (Fig. 3*A* and Supplementary Table 2). A driver mutation was detected in tissue alone in two patients (4%); both harbored an *EML4-ALK* fusion. Combined use of tissue and plasma sequencing identified a therapeutically informative driver mutation in 62% of patients, whereas tissue alone detected a driver mutation in 51%. Concordance between tissue and plasma sequencing was 90%.

The median time (interquartile range [IQR]) from diagnostic biopsy to first medical oncology visit was similar between cohorts (cohort 1: 12 d [IQR: 7–19]; cohort 2: 14 d [IQR: 9–21]; *p* = 0.32, Mann-Whitney). NGS results were available before the first medical oncology visit more frequently in cohort 1 (85% versus 9%, *p* < 0.0001, Pearson’s chi-square; Fig. 3*B*). For patients with nonsquamous NSCLC, reflex tissue NGS was completed for 85% of patients in both cohorts, with 15% of patients having insufficient quantity or quality of tissue DNA for NGS. Turnaround time for plasma NGS was shorter compared with tissue (8 versus 26 d, *p* < 0.0001, Mann-Whitney). Among the 46 patients in cohort 1 with nonsquamous NSCLC evaluated by a medical oncologist, a higher percentage of patients received specific treatment recommendations at the first visit compared with the 54 patients in cohort 2 (74% versus 46%, *p* = 0.005, Pearson’s chi-square; Fig. 3*C*). In these patients, the median time-to-treatment was shorter in cohort 1 compared with cohort 2 (12 [IQR: 8–20] versus 20 d [IQR: 13–24], *p* = 0.003, Mann-Whitney), with an even shorter time-to-treatment when limited to patients identified to have a specific driver mutation (10 [IQR: 8–15] versus 19 d [IQR: 13–22], *p* = 0.001, Mann-Whitney; Fig. 3*D*).

## Discussion

These results provide evidence supporting a multidisciplinary approach for guideline-recommended plasma NGS earlier in the diagnostic evaluation of patients with advanced NSCLC. Patients with NSCLC with plasma genotyping performed at the time of initial diagnostic biopsy had a significantly shorter time-to-treatment compared with a historical cohort undergoing reflex tissue NGS. This effect was more pronounced in patients with a therapeutically informative driver mutation identified by plasma genotyping, as therapeutic decisions can be made on receiving sequencing results. This approach of obtaining plasma NGS at the time of diagnostic biopsy may inform therapeutic decisions in a significant percentage of cases, as oncogenic drivers are detected in approximately 58% to 64% of patients with NSCLC, similar to our population.^8^^,^^9^ In our historical cohort, only 9% of the patients had NGS results available at the first medical oncology appointment and 46% of these patients received a specific treatment recommendation. In the prospective cohort, 85% of the patients had NGS results available at the first visit, with 74% receiving an NGS-informed treatment recommendation suggesting that plasma NGS at the time of biopsy enabled the timely delivery of genomic results so that treatment recommendations could be made expeditiously and on the basis of sequencing results.

Ensuring patients with newly diagnosed advanced NSCLC receive timely and guideline-concordant biomarker testing remains a significant clinical challenge. Molecular profiling, whether through tissue or plasma-based genotyping, is often initiated at the first oncology visit leading to treatment delays, inefficiencies, and uncertainties when discussing management options; some patients may not be able to wait for these results without clinical deterioration. Although the impact of this approach demonstrating a shorter time-to-treatment of 8 days may be modest, this outcome is patient-centered and likely meaningful to both patients and treating oncologists. In addition, these results were compared with reflex tissue sequencing which is not available in most practices and further studies are warranted to evaluate this approach in a variety of clinical practice settings. Here, we provide proof-of-concept for a multidisciplinary and patient-centered approach to implementing plasma genotyping earlier in the diagnostic evaluation of patients with suspected advanced NSCLC to ensure the timely delivery of personalized medicine.

Our study does have certain limitations. It is a single-center, nonrandomized study, conducted at a tertiary academic center. The success of this approach depends on the reliable and timely communication of NGS results to treating physicians, which may be easier when all aspects of care are delivered at the same institution. In addition, 15% of enrolled subjects ultimately did not have NSCLC (eight SCLC, one breast cancer, and one malignant epithelioid neoplasm). Although rapid on-site cytology was not used in this study, the use of this practice to confirm a nonsmall cell histology before sending plasma for sequencing may reduce the number of tests that are unlikely to inform clinical decision-making. Finally, although this approach is patient centered, which could alleviate anxiety of awaiting genomic results and reducing treatment delays, the cost of performing parallel sequencing of plasma and tissue warrants further investigation.

Our results reveal that plasma-based sequencing performed at the time of diagnostic biopsy in patients with suspected advanced NSCLC is associated with decreased time-to-treatment compared with reflex tissue genotyping. Further prospective studies are warranted to validate these findings and determine whether this approach is associated with improved patient outcomes and satisfaction.

## CRediT Authorship Contribution Statement

**Jeffrey C. Thompson, C. Aggarwal, E. Carpenter:** Conceptualization, Methodology.

**Jeffrey C. Thompson, C. Aggarwal, J. Wong, M. Andronov, D. M. Dibardino, C. T. Hutchinson, K. C. Ma, A. Lanfranco, E. Moon, A. R. Haas, A. P. Singh, C. A. Ciunci, M. Marmarelis, C. D’Avella, J. V. Cohen, J. M. Bauml, R. B. Cohen, C. J. Langer:** Investigation.

**Jeffrey C. Thompson, C. Aggarwal, E. Carpenter, J. Wong, V. Nimgaonkar, M. Andronov, W. Hwang:** Data curation.

**Jeffrey C. Thompson, C. Aggarwal, E. Carpenter, J. Wong, V. Nimgaonkar, A. Vachani, W. Hwang:** Formal Analysis.

**Jeffrey C. Thompson, C. Aggarwal, J. Wong, V. Nimgaonkar, W. Hwang, M. Andronov, D.M. Dibardino, C. T. Hutchinson, K. C. Ma, A. Lanfranco, E. Moon, A. R. Haas, A. P. Singh, C. A. Ciunci, M. Marmarelis, C. D’Avella, J.V. Cohen, J. M. Bauml, R. B. Cohen, C. J. Langer, A. Vachani, E. L. Carpenter:** Manuscript writing, Final approval of manuscript.
