# Supplementary material for: Plasma Genotyping at the Time of Diagnostic Tissue Biopsy Decreases Time-to-Treatment in Patients With Advanced NSCLC—Results From a Prospective Pilot Study
Source: JTO Clin Res Rep. 2022 Mar 8;3(4):100301. doi: 10.1016/j.jtocrr.2022.100301 (PMC8980884; doi:10.1016/j.jtocrr.2022.100301)
Supplement: Supplemental Table 1 [file mmc1.pdf]

eTable 1.

Characteristics	Cohort 1 (n=55)	Cohort 2 (n=55)	p-value[1]
**Age (Range)**	67 (41-87)	67 (36-82)	0.81
**Sex**			
**Female**	34 (62%)	24 (44%)	0.06
**Male**	21 (38%)	31 (56%)	
**Race**			0.09
**White**	32 (58%)	42 (76%)	
**Black**	15 (27%)	9 (16%)	
**Asian**	5 (9%)	3 (5%)	
**Other**	3 (6%)	0 (0%)	
**Unknown**	0 (0%)	1 (2%)	
**Smoking History**			0.84
**Never**	11 (20%)	11 (20%)	
**Current**	8 (15%)	6 (11%)	
**Former**	36 (65%)	38 (69%)	
**Biopsy Method**			0.03
**Bronchoscopy**	47 (85%)	37 (67%)	
**Other**	8 (15%)	18 (33%)	
**NSCLC Histology**			0.06
**Non-squamous**	48 (87%)	54 (98%)	
**Squamous**	7 (13%)	1 (2%)	
**Stage**			0.50
**IIIB**	2 (3.6%)	--	
**IV**	53 (96%)	55 (100%)	
**Treatment Class**			0.20
**Tyrosine Kinase Inhibitor**	17 (31%)	10 (18%)	
**Immunotherapy + Chemotherapy**	22 (40%)	26 (47%)	
**Immunotherapy**	7 (13%)	7 (13%)	
**Chemotherapy**	3 (5%)	9 (11%)	
**None (Hospice, Death)**	6 (11%)	3 (5%)	

[1]Pearson's Chi-squared test, Fisher's exact test for categorial variables; Wilcoxon rank sum test for continuous variables.
