# Supplementary material for: Plasma Genotyping at the Time of Diagnostic Tissue Biopsy Decreases Time-to-Treatment in Patients With Advanced NSCLC—Results From a Prospective Pilot Study
Source: JTO Clin Res Rep. 2022 Mar 8;3(4):100301. doi: 10.1016/j.jtocrr.2022.100301 (PMC8980884; doi:10.1016/j.jtocrr.2022.100301)
Supplement: Supplemental Table 2 [file mmc2.pdf]

eTable 2.

Gene	Cohort 1 Driver Mutations	Cohort 2 Driver Mutations
KRAS	(6) G12C<br>(3) G12V<br>(1) G12A<br>(1) G12I<br>(1) G12D<br>(1) G12S	(9) G12C<br>(5) G12D<br>(3) G12V<br>(2) G12A<br>(1) G12S
ERBB2 (HER2)	(1) V777L<br>(1) Exon 20 insertion	(1) Exon 20 insertion
EGFR	(6) L858R<br>(6) Exon 19 deletion<br>(1) Exon 20 insertion	(5) L858R<br>(5) Exon 19 deletion<br>(1) Exon 20 insertion
ALK	(1) EML4 Exon 20/Exon 20 fusion<br>(1) EML4 Exon 6/Exon 20 fusion	----
RET	(1) KIF5B Fusion	----
BRAF	(1) V600E	(2) V600E
MET	(2) Exon 14 skipping	(1) Exon 14 skipping

Genes evaluated for driver mutations included *ALK, BRAF, EGFR, ERBB2, KRAS, MET, RET, ROS1, and NTRK*. Total driver mutations detected: Cohort 1 (n=34), Cohort 2 (n= 35)
